# Impact of Collection Volume and DNA Extraction Method on the Detection of Biomarkers and HPV DNA in First-Void Urine

**DOI:** 10.3390/molecules26071989

**Published:** 2021-04-01

**Authors:** Laura Téblick, Severien Van Keer, Annemie De Smet, Pierre Van Damme, Michelle Laeremans, Alejandra Rios Cortes, Koen Beyers, Vanessa Vankerckhoven, Veerle Matheeussen, Renee Mandersloot, Arno Floore, Chris J. L. M. Meijer, Renske D. M. Steenbergen, Alex Vorsters

**Affiliations:** 1 Centre for the Evaluation of Vaccination (CEV), Vaccine & Infectious Disease Institute (VAXINFECTIO), Faculty of Medicine and Health Sciences, University of Antwerp, 2610 Wilrijk-Antwerp, Belgium; severien.vankeer@uantwerpen.be (S.V.K.); Annemie.desmet@uantwerpen.be (A.D.S.); pierre.vandamme@uantwerpen.be (P.V.D.); alex.vorsters@uantwerpen.be (A.V.); 2 Novosanis nv, 2110 Wijnegem, Belgium; Michelle.Laeremans@vito.be (M.L.); alejandra.rioscortes@novosanis.com (A.R.C.); koen.beyers@novosanis.com (K.B.); vanessa.vankerckhoven@novosanis.com (V.V.); 3 Department of Microbiology, Antwerp University Hospital (UZA), 2650 Edegem-Antwerp, Belgium; Veerle.Matheeussen@uantwerpen.be; 4 Department of Medical Microbiology (LMM), Vaccine & Infectious Disease Institute (VAXINFECTIO), Faculty of Medicine and Health Sciences, University of Antwerp, 2610 Wilrijk-Antwerp, Belgium; 5 Department of Medical Biochemistry, Faculty of Pharmaceutical, Biomedical and Veterinary Sciences, University of Antwerp, 2610 Wilrijk-Antwerp, Belgium; 6 Self-Screen B.V, 1098 RX Amsterdam, The Netherlands; r.mandersloot@gmail.com (R.M.); an.floore@self-screen.nl (A.F.); cjlm.meijer@self-screen.nl (C.J.L.M.M.); 7 Amsterdam UMC, Pathology, Cancer Center Amsterdam, Vrije Universiteit Amsterdam, 1081 HV Amsterdam, The Netherlands; r.steenbergen@amsterdamumc.nl

**Keywords:** first-void urine, human papillomavirus, HPV, self-sampling, cervical cancer, extractions, genotyping, optimization

## Abstract

The potential of first-void (FV) urine as a non-invasive liquid biopsy for detection of human papillomavirus (HPV) DNA and other biomarkers has been increasingly recognized over the past decade. In this study, we investigated whether the volume of this initial urine stream has an impact on the analytical performance of biomarkers. In parallel, we evaluated different DNA extraction protocols and introduced an internal control in the urine preservative. Twenty-five women, diagnosed with high-risk HPV, provided three home-collected FV urine samples using three FV urine collection devices (Colli-Pee) with collector tubes that differ in volume (4, 10, 20 mL). Each collector tube was prefilled with Urine Conservation Medium spiked with phocine herpesvirus 1 (PhHV-1) DNA as internal control. Five different DNA extraction protocols were compared, followed by PCR for GAPDH and PhHV-1 (qPCR), HPV DNA, and HBB (HPV-Risk Assay), and ACTB (methylation-specific qPCR). Results showed limited effects of collection volume on human and HPV DNA endpoints. In contrast, significant variations in yield for human endpoints were observed for different DNA extraction methods (*p* < 0.05). Additionally, the potential of PhHV-1 as internal control to monitor FV urine collection, storage, and processing was demonstrated.

## 1. Introduction

The potential of first-void (FV) urine as a suitable, non-invasive liquid biopsy for detection of human papillomavirus (HPV) DNA and other biomarkers to detect high-grade cervical abnormality, has already been reported [1,2,3,4,5,6,7,8]. High correlates between urinary HPV DNA and cervical infections have been established and studies have confirmed the presence of HPV specific antibodies in FV urine [9,10,11,12,13,14,15,16]. Urine is considered the preferred choice of self-sampling compared to the currently available self-sampling methods used for screening; brush, lavage, spatula, swab and tampon [17,18,19,20,21,22,23,24]. Especially during the current COVID-19 (Coronavirus Disease 2019) pandemic, at-home self-sampling will provide a more accessible means for screening or follow-up than attending health care facilities. However, to use FV urine as custom liquid biopsy for cervical cancer screening, potential optimization of the sample collection and DNA extraction workflow should be considered.

The rationale behind the use of FV urine is based on the fact that the initial stream of urine – first-void– washes away secretions that accumulate between the small labia and around the urethra opening, including mucus and debris from exfoliated cells from the vagina, cervix, and uterus [25]. This explains why the first fraction of a urine void contains significantly more HPV DNA than the subsequent part [12]. However, the optimal volume of the initial urine stream to provide the highest concentration of biomarkers of interest has not been established yet. The currently used FV urine collection device (Colli-Pee^®^, Novosanis, Wijnegem, Belgium), prefilled with Urine Conservation Medium (UCM), collects the first fraction of urine in a total volume of 20 mL (one part UCM and two parts FV urine). The UCM buffer has already shown to positively influence detectable HPV DNA by avoiding degradation of cell-free DNA by nucleases [1]. The ideal FV urine volume would provide optimal concentration of HPV and human DNA for accurate detection of clinically relevant high risk (HR)-HPV infections. In addition, use of customized tubes, like the routinely used 10 mL tubes, could allow fully automated high-throughput testing.

An internal process control is another important factor to validate a sample for analysis. This internal control needs to be able to monitor the presence of sufficient preservative, demonstrate that the extraction was well performed, and show that no degradation of DNA/biomarker of interest took place. Earlier research in our lab showed that human DNA is not entirely sufficient as an internal process control since inappropriately stored samples still had detectable human DNA, while the HPV DNA test became false negative [1]. In addition, the amount of human DNA is very variable between samples/individuals. As detection of cell-free viral DNA is also essential, the most suitable internal control would be DNA from a whole virus preparation.

To date, there are numerous adequate DNA extraction methods on the market, each having its own advantages and limitations [2]. However, it has not yet been investigated whether the extraction method used in combination with different FV urine volumes influences the yield of recovered human DNA, HPV DNA, and other biomarkers of interest. The evaluation of the distinct DNA extraction methods and FV urine collection volumes in combination with the introduction of an internal process control offers additional opportunities for the use of FV urine as a suitable sample for the detection of HPV. 

In many European Union (EU) countries, cervical cancer screening is based on cytology. This requires a physician-taken cervical scrape and is challenged by low sensitivity (30% false-negatives) and high non-attendance (63% EU coverage) [26]. The combination of a FV urine collector and an integrated diagnostic approach for both HPV detection and triage after an HPV positive result to identify women with cervical cancer or precursor lesions could maximize screening participation.

In this study, we investigated whether the FV urine collection volume affects the detection of human and viral endpoints (i.e., glyceraldehyde 3-phosphate dehydrogenase (GAPDH), β-actin (ACTB), β-globin (HBB), HPV) by comparing three different FV urine collection volumes (4, 10, and 20 mL). In addition, we evaluated different DNA extraction protocols and introduced a universal non-human control (i.e., DNA from phocine herpesvirus 1 (PhHV-1) [27,28]) in the urine preservative to monitor sample collection, transport, storage, and DNA extraction.

## 2. Results

### 2.1. Sample Collection

**Figure 1:**
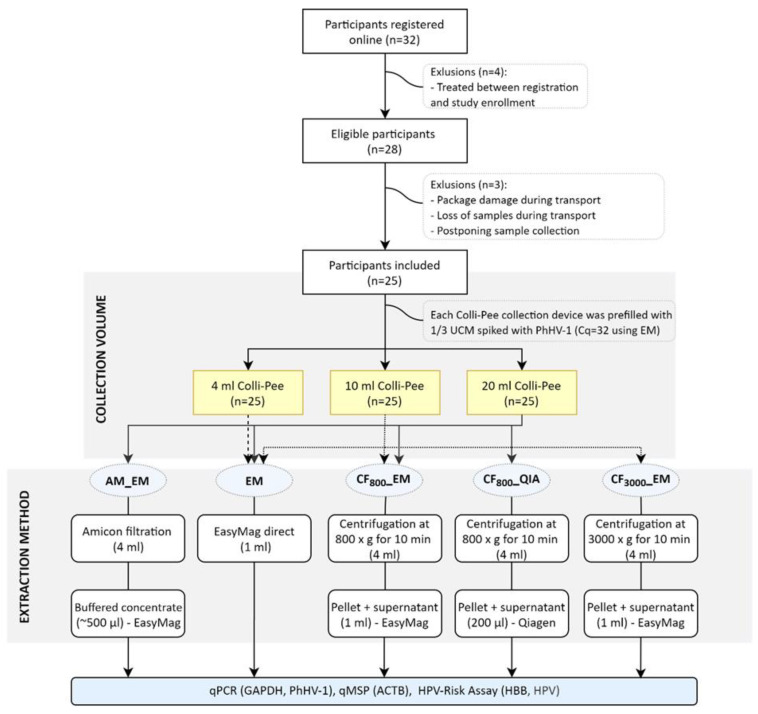
Flow diagram of the study; 32 women subscribed online, whereof 28 eligible women were included. Each woman received a study package at home as described. From those 28 women, three women were excluded (reasons for exclusion listed in the figure). All 25 included women collected three consecutive samples using three different Colli-Pee^®^ urine devices with collector tubes that differ in size. On the same day of collection (or at the latest, one day after collection), the urine samples were sent back to the lab via postal mail where they were aliquoted and stored at −80 °C prior to further analysis. On each sample, DNA was extracted using the EM. On the samples collected with Colli-Pee^®^ 20 mL and 10 mL, three and two other DNA extraction methods were performed, respectively. All DNA extracts were analyzed for GAPDH and phocine herpesvirus 1 (PhHV-1) using an in-house qPCR. In addition, samples were analyzed using the human papillomavirus (HPV)-Risk Assay and methylation-specific qPCR at Self-Screen B.V. n; number, EM; extraction method, SN; supernatant.

Three samples, all different volumes at random order, were received from 25 women aged between 19 and 62 years old (overview in Figure 1). The median age in the cohort was 31 (interquartile range (IQR): 27–35). Median (IQR) estimated collection volumes were 4.1 (3.8–4.4) mL, 9.8 (9.4–10.3) mL, and 20.3 (19.8–20.8) mL with collector tubes estimated to collect 4 mL, 10 mL, and 20 mL, respectively. The three samples were collected within a median (IQR) time interval of 3h 2min (2:30–4:26). Two women collected one sample on another day than the other two samples. Data for GAPDH, PhHV-1, ACTB, HBB, and HPV DNA was generated from 3 × 25 FV urine samples.

### 2.2. Evaluation of PhHV-1 as Internal Process Control

**Figure 2:**
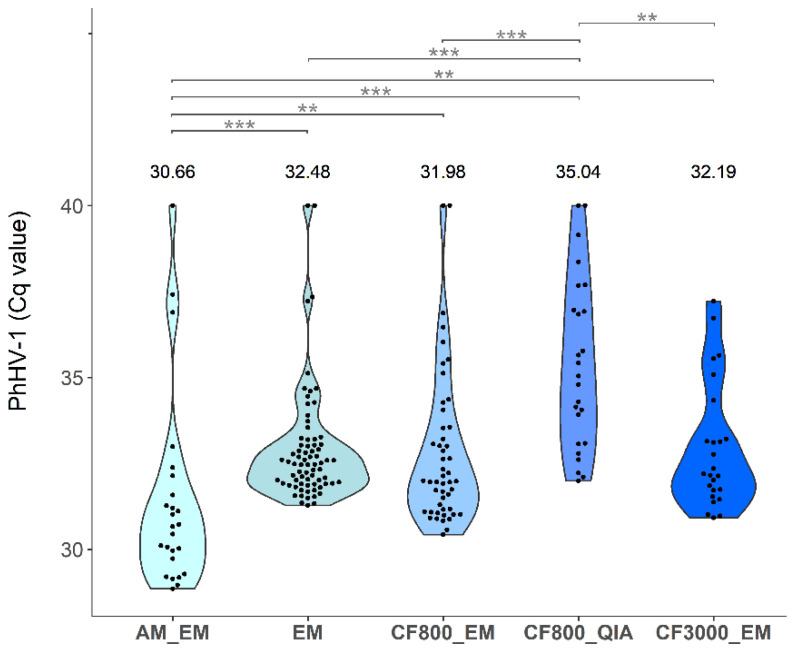
Violin plot of qPCR Cq values for PhHV-1 for all 25 included samples. EM was used for all extraction volumes, CF_800__EM for both 10 mL and 20 mL, AM_EM, CF_800__QIA only for 20 mL, and CF_3000__EM only for 10 mL. Calculated cut-offs for each extraction method are; AM_EM: 36.26, EM: 35.83, CF_800__EM: 36.40, CF_800__QIA: 40.00, CF_3000__EM: 3 5.81. Significance levels are represented in the figure by an asterisk (** *p* < 0.01; *** *p* < 0.001).

**Figure A1:**
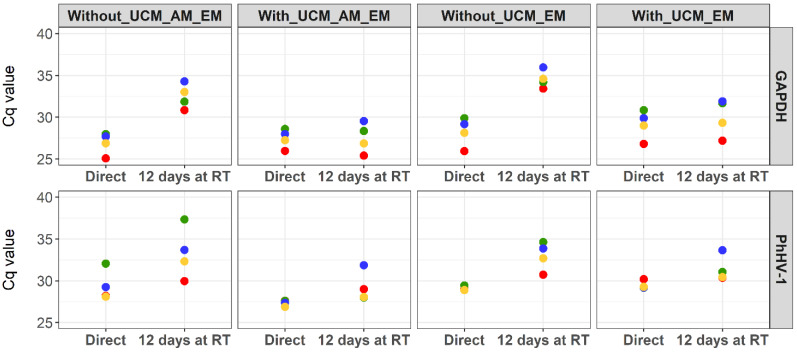
Results for GAPDH (**upper** figures) and PhHV-1 (**lower** figures) on 4 FV urine samples. Each ID has a unique color mating its Cq values. Samples were processed directly (Direct) and after 12 days of storage at room temperature. Presence of UCM in the samples is referred to as either “With_UCM” or “Without_UCM”. The use DNA Extraction method is referred to as well; AM_EM or EM. No PhHV-1 result was obtained for ID3 at time point 0 for category With_UCM_AM_EM due to pipetting error. UCM; Urine Conservation Medium, EM; EasyMag, AM; Amicon filtration.

**Figure A2:**
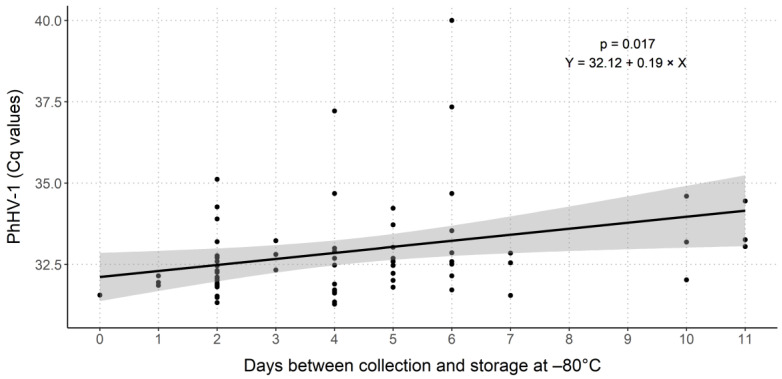
Spreading and correlation curve of PhHV-1 Cq values of DNA extracted with EM over timespan between collection of the urine sample and storage at −80 °C. A linear model was used to evaluate this effect. For each extra day between FV urine collection and storage at −80 °C, the Cq value increased with 0.19. This effect was significant. No such significant effect was seen for all other human endpoints.

Internal control quantification cycle (Cq) value results of the 25 women included in the study were generated by qPCR (Figure 2). Since spiked PhHV-1 concentrations in the different FV urine collection volumes were identical and no significant differences in Cq values were observed between the different collection volumes when using the same extraction method (*p* ≥ 0.36), cut-offs were calculated for each DNA extraction method separately, but on all volumes combined (cut-offs; AM_EM: 36.26, EM: 35.83, CF_800__EM: 36.40, CF_800__QIA: 40.00, CF_3000__EM: 35.81). Comparing three different collection volumes using EM and four extraction methods on 20 mL, three IDs (1, 8, and 19) exceeded the calculated cut-offs. Comparing two collection volumes using CF_800__EM and three extraction methods on 10 mL, also three samples (8, 15, and 19) exceeded the calculated cut-offs. Since the sample size is too low to validate the exploratory defined cut-off, all samples were included in further analyses. The distribution of Cq values varied between different extraction methods. A linear model was used to investigate the effect of time (days) between sample collection and freezing of aliquoted samples on the DNA yield of PhHV-1, GAPDH, HBB, and ACTB using extraction method EM (Figure A1). For none of the human endpoints, a significant effect was found (all *p* ≥ 0.05). However, there was a significant effect of time between collection and freezing on the PhHV-1 Cq values (*p* = 0.017). For each extra day between FV urine collection and storage at −80 °C for samples extracted with EM, the median Cq value increased by 0.19 (Figure A2).

### 2.3. Comparison of DNA and Biomarker Yields between FV Urine Collection Volumes

#### 2.3.1. Human Endpoints

**Figure 3:**
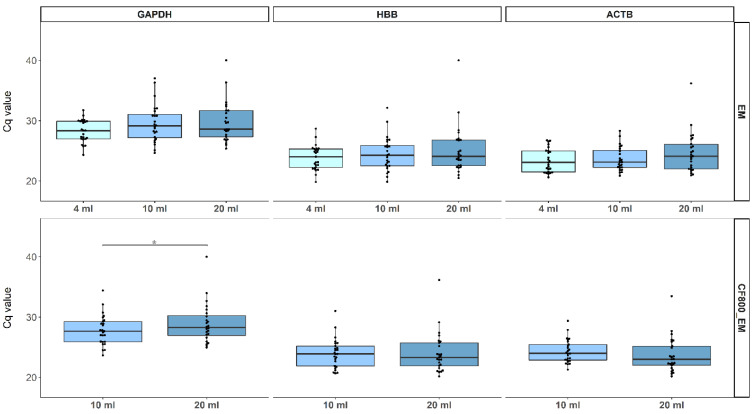
Boxplots representing the Cq values of GAPDH, HBB, and ACTB for each evaluated collection volume using EM (upper figures) and CF_800__EM (lower figures). Individual Cq values are represented as dots. All data were analyzed using the non-parametric Wilcoxon signed-rank testing. Significant differences are represented in the figure by an asterisk (*p* < 0.05).

**Figure A3:**
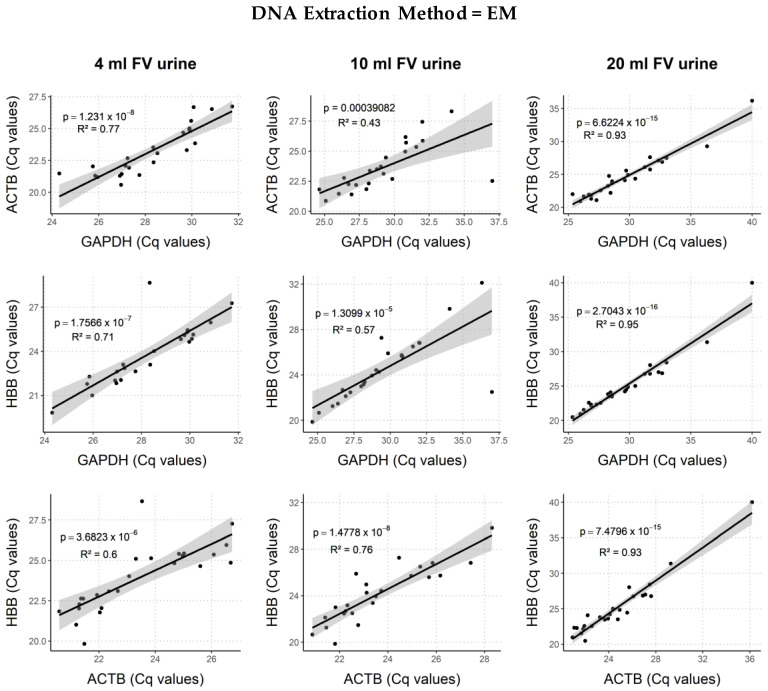
Correlation curves for all human endpoint combinations on DNA extracted using EM for 4, 10, and 20 mL FV urine collection volumes. High risk (HR)-HPV positive samples are marked grey. All correlations are highly significant. R² values are highest for 20 mL and lowest for 10 mL samples.

**Figure A4:**
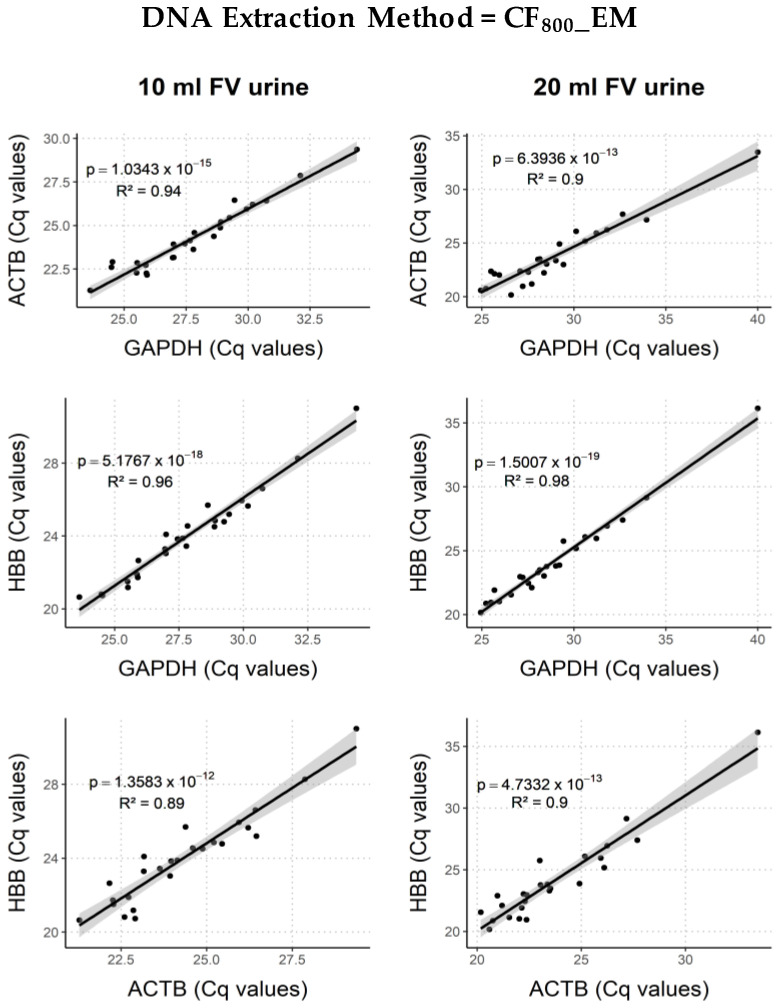
Correlation curves for all human endpoint combinations on DNA extracted using CF_800__EM for 10 and 20 mL FV urine collection volumes. HR-HPV positive samples are marked grey. All correlations are highly significant, and R² values are similar for both volumes.

PCR Cq value results, inversely proportional to concentrations, for GAPDH, HBB, and ACTB were generated for all three FV urine collection volumes using extraction method EM and on 10 mL, and 20 mL samples using CF_800__EM. Results are presented as boxplots (Figure 3). Using EM, we observed no significant differences between the three FV urine collection volumes for all human endpoints (GAPDH: *p* ≥ 0.07, ACTB: *p* ≥ 0.40, HBB: *p* ≥ 0.28) (Figure 3). In addition, similar and all highly significant (*p* ≤ 3.9 × 10^−4^) Pearson correlations were found between all three housekeeping genes; GAPDH, HBB, and ACTB, for all collection volumes (Figure A3). The timing of the three FV urine collections did also not have a significant effect on the DNA Cq value for all three human endpoints (data not shown). For CF_800__EM, there was no significant Cq difference for HBB (*p* = 0.85) and ACTB (*p* = 0.083) between 10 and 20 mL collections. We did observe significant differences in Cq for GAPDH (*p* = 0.033). Here median Cq value for 20 mL (28.3) was higher than for 10 mL (27.6). However, the same Pearson correlation trend between all housekeeping genes was seen for CF_800__EM (*p* ≤ 1.36 × 10^−12^) compared to EM (Figure A4). 

#### 2.3.2. Viral Endpoints

HPV DNA results for different volumes were compared using the DNA extraction method EM (Table 1). Samples were classified as HR-HPV positive when HR-HPV DNA was present. Here, no samples showed presence of HPV-16 or -18 DNA. However, in total 37% (28/75) of the DNA samples extracted with EM and 44% (23/50) with CF_800__EM contained HPV DNA of an HR-HPV type included in the HPV-Risk assay. Using EM, FV urine samples collected with the 4 mL device showed 32% (8/25) HR-HPV positivity, the 10 mL device 44% (11/25), and the 20 mL 36% (9/25). No significant differences were found (*p* ≥ 0.14). Concordance of the HR-HPV positivity results over the different FV urine collection volumes are K_4/10_ = 0.58 and K_4/20_ = 0.91, K_10/20_ = 0.67. For CF_800__EM, FV urine samples collected with the 10 mL device showed 52% (13/25) HR-HPV positivity and the 20 mL 40% (10/25). Here, Cohen’s Kappa value is 0.60. While not all volumes gave HR-HPV positive results, the Cq values were high (34.71; 28.89–38.78) in the sample where HR-HPV DNA was detected.

### 2.4. Comparison of DNA and Biomarker Yields between DNA Extraction Methods

#### 2.4.1. Human Endpoints

**Figure 4:**
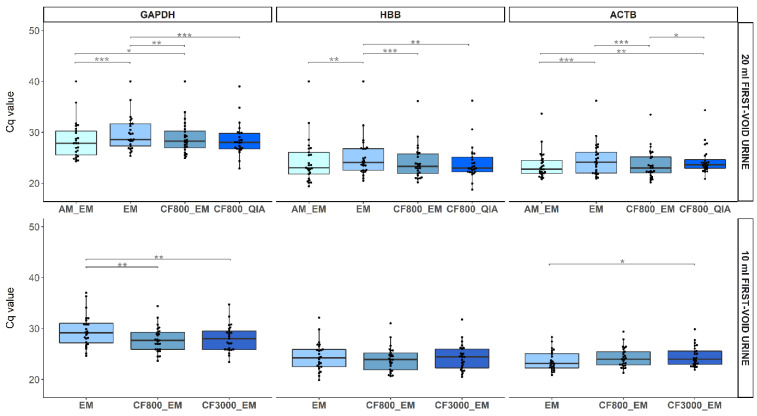
Boxplots representing the Cq value of GAPDH, HBB, and ACTB for each DNA extraction method on both 20 mL samples (upper figures) and 10 mL sample volume (lower figures). Individual Cq values are represented as dots. All data were analyzed using the non-parametric Wilcoxon signed-rank testing. Significance levels are represented in the figure by an asterisk (* *p* < 0.05; ** *p* < 0.01; *** *p* < 0.001).

**Figure A5:**
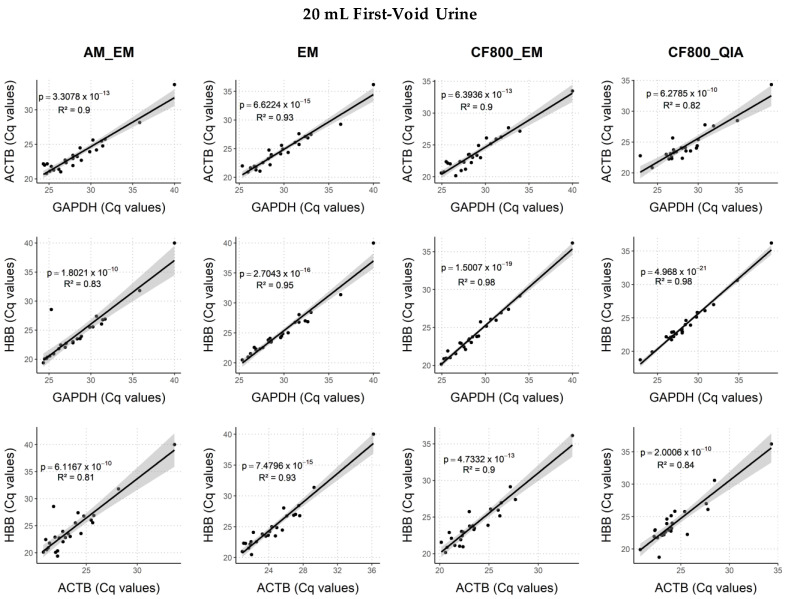
Correlation curves for all human endpoint combinations on 20 mL FV urine samples extracted with AM_EM, EM, CF_800__EM, and CF_800__QIA. HR-HPV positive samples are marked grey. All correlations are highly significant.

**Figure A6:**
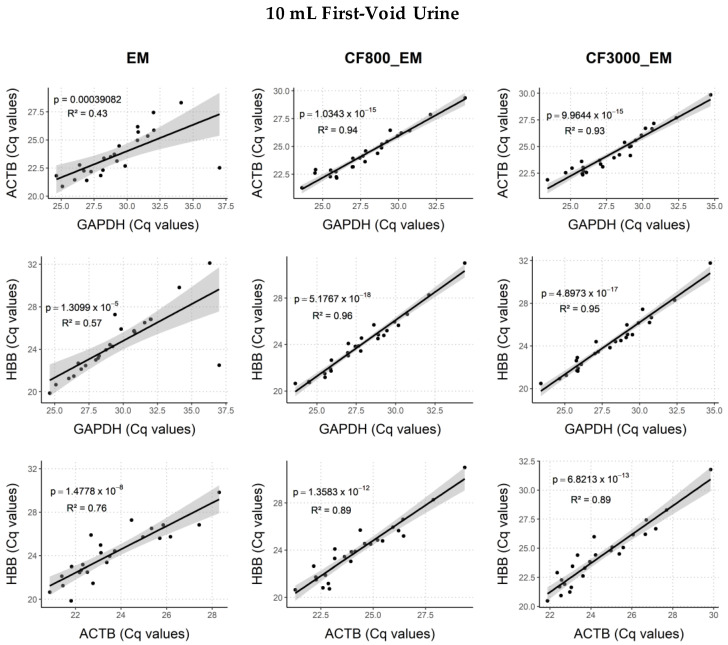
Correlation curves for all human endpoint combination on 10 mL FV urine samples extracted with EM, CF_800__EM, and CF_3000__EM. HR-HPV positive samples are marked grey. All correlations are highly significant. Using the method EM, R² values are lower than CF_800__EM, and CF_3000__EM.

DNA yield results for GAPDH, HBB, and ACTB on the different DNA extraction methods were generated and presented as boxplots (Figure 4). Comparing four DNA extraction methods (AM_EM, EM, CF_800__EM, and CF_800__QIA) used on 20 mL FV urine samples, GAPDH Cq values were significantly higher for EM in comparison to all other methods (*p* ≤ 0.12 × 10^−3^). In addition, AM_EM had a lower Cq value for GAPDH than CF_800__EM (*p* = 0.04). The HBB Cq values for EM were significantly higher than for AM_EM (*p* = 0.21 × 10^−2^), CF_800__EM (*p* = 0.26 × 10^−3^), and CF_800__QIA (*p* = 0.79 × 10^−2^)). The quantitative Methylation-Specific PCR (qMSP) results for ACTB showed significantly lower yield for EM (*p* = 0.16 × 10^−3^) and CF_800__QIA (*p* = 0.52 × 10^−2^) compared to results for AM_EM. Moreover, CF_800__EM had significantly lower Cq-values than EM (*p* = 7.40 × 10^−5^) and CF_800__QIA (*p* = 0.01), but no significant difference was observed between CF_800__EM and AM_EM (*p* = 1.00). There was a significant Pearson correlation between all human endpoint combinations (*p* ≤ 1.80 × 10^−10^) for all extraction methods (Figure A5). Since extraction method CF_800__EM showed promising results and a smaller collection volume has transport and storage advantages, the remaining volume of the 10 mL FV urine sample was used to compare two centrifugation speeds (CF_800__EM and CF_3000__EM). In total, three different DNA extraction methods (EM, CF_800__EM, and CF_3000__EM) were compared using 10 mL FV urine samples. Here, GAPDH Cq values were significantly lower, using CF_800__EM or CF_3000__EM compared to EM (*p* ≤ 0.35 × 10^−2^). The HBB Cq values showed no significant differences for all methods (*p* ≤ 0.08). Results for ACTB showed significantly higher yield for EM compared to CF_3000__EM (*p* = 0.016). Other observed variations were not significant (Figure 4). For all three extraction methods, there was a significant Pearson correlation between all human endpoint combinations (*p* ≤ 3.91 × 10^−4^) (Figure A6). For the 4 mL FV urine samples, only extraction method EM was used since there was not enough volume left to perform additional extractions. 

#### 2.4.2. Viral Endpoints

Results for different extraction methods were evaluated on 20 mL and 10 mL samples (Table 1). No samples showed the presence of HPV16 or -18 DNA. HR-HPV infections were found in 39% (39/100) of the 20 mL FV urine DNA extracts. DNA extracted with AM_EM showed 44% (11/25) HR-HPV positivity, with EM 36% (9/25), with CF_800__EM 40% (10/25), and with CF_800__QIA 36% (9/25). No significant differences were found (*p* ≥ 0.09). Here, the Fleiss Kappa value is 0.87. In total 48% (36/75) of the 10 mL FV urine DNA extracts contained HPV DNA of an HR-HPV type included in the HPV-Risk assay. DNA extracted with EM showed 44% (11/25) HR-HPV positivity, CF_800__EM 52% (13/25), and CF_3000__EM 48% (12/25). Concordance of the HR-HPV positivity results over the different DNA extraction methods on 10 mL samples was 0.78. Whenever not all methods gave HR-HPV positive results, the Cq values were high (36.39; 30.83–39.24) in the extracts where HR-HPV DNA was detected.

## 3. Discussion

The use of FV urine as a self-sample for HPV DNA testing has already proven promising and high correlates between urinary HPV DNA and cervical infections have been established [3]. Combining this non-invasive and user-friendly sample with a tailored molecular assay for the detection of pre-cancer biomarkers in urine [8,29,30,31] could provide an alternative for the current non-attendees of screening programs and ultimately the entire screening population. We studied several sample collection and extraction workflows (i.e., internal control, collection volume, DNA extraction method) to investigate if we could improve the performance of FV urine as a liquid biopsy.

### 3.1. Internal Process Control

As part of the optimization process, we introduced phocine herpesvirus type 1 (PhHV-1) as internal process control in the UCM and observed degradation in PhHV-1 DNA when samples were stored for 12 days at ambient temperature (Figure A1) whereas this was not seen for GAPDH when samples were stored with UCM. This difference can be due to the fact that GAPDH concentrations are subsidiary to interpersonal variation and PhHV-1 DNA is spiked in a constant concentration. PhHV-1 Cq values furthermore linearly increased with an average of 0.19 Cq per additional day passed between sample collection and storage at −80 °C in this specific sample set of 75 FV urine extracts (extraction method EM on all three collection volumes), confirming its potential as an internal control for sample storage. The selection of a cell-free internal control is based upon previous studies where it was shown that non-cell associated DNA in FV urine samples is most vulnerable to degradation [1,2]. Since the buffer is spiked with PhHV-1, not detecting this control will indicate inappropriate sample collection. Due to PhHV-1 DNA being cell-free, and thus not able to pellet, it is impossible to combine this control with an extraction method that only uses the pellet for qPCR (CF_800__QIA). Small fluctuations between PhHV-1 Cq values within the same DNA extraction method can be due to different sample composition and storage conditions [2,32]. The internal control cut-off calculated in this pilot sample set will be evaluated in a larger external validation set (CASUS, clinicaltrials.gov: NCT04530201). Combining the UCM [1] with an internal process control will allow laboratories to set up a strict sample control quality system, confirming the presence of the preservative and absence of substantial nucleic acid degradation.

### 3.2. FV Urine Collection Volume

The most widely used method for urine collection to screen for infectious agents or other biomarkers is the use of a urine collection cup. However, these cups are not designed to collect the first urine fraction, making it more difficult to confidently collect the initial urine stream with high concentrations of certain biomarkers [12,32]. Colli-Pee^®^ is a commercially available device that collects a standardized, pre-defined volume of FV urine without the need to interrupt the urine current. With a view toward volume optimization, different FV urine collection devices collecting a fixed volume of the initial urine stream were evaluated. Results of the experimental arms compared in this study show limited differences between the FV urine collection volumes for all human endpoints using EM. The human endpoints evaluated within this study represent the sample quality for both HPV DNA testing and biomarker testing and are therefore important factors to evaluate. Since HPV DNA detection using the 20 mL collection device already provided promising analytical and clinical accuracy results [1,17,32,33,34]. These additional results also support the potential of the 4- and 10- mL collection volume. For CF_800__EM, there was a significant difference between 10- and 20-mL samples for GAPDH. When comparing HPV DNA Cq values, more HR-HPV infections were detected using Colli-Pee^®^ for the collection of 10 mL compared to 4- and 20-mL. Based on these results, we can conclude that collecting only 10 ml could provide an increased sample quality for the detection of HPV DNA. In addition, we hypothesize that 4 mL of FV urine (approximately 2.33 mL when taking the buffer into account), is not sufficient to wash away all secretions that have accumulated between the urethra and small labia. Since there is a clear biological link between HPV infection and cervical pre-cancer and cancer, the highest HPV DNA concentration will also be a good representation for the highest amount of biomarkers, including methylation markers [35,36,37,38]. Besides, the smaller collector tube of the 10 ml in contrast to the 20 mL device has advantages in transport (by, e.g., postal mail), storage, and high-throughput testing if a fully integrated nucleic acid extraction and testing platform is used.

### 3.3. DNA Extraction Method

To date, there has not been a comparison between DNA extraction methods in combination with different FV urine collection volumes. Since the DNA extraction can most likely influence the concentration of a certain biomarker, we compared five different extraction methods overall, including a direct comparison of four methods on 20 mL FV urine samples. Almost all differences in Cq value can directly be linked to the difference in implemented volumes. Variation in centrifugal forces did not cause different DNA yields (CF_800__EM vs. CF_3000__EM). For both human and viral endpoints using only the pellet for extraction (CF_800__QIA) gave similar DNA yields in comparison to other methods. However, this extraction method is not compatible with the selected internal control given that cell-free viral DNA is not concentrated by the pellet. Considering all factors, including costs and amount of laboratory work, which are higher for AM_EM and CF_800__QIA and lower yields of human and viral endpoints for EM; preference is given to CF_800__EM.

### 3.4. Limitations of the Study

A number of limitations regarding this study need to be acknowledged. First, the relatively small sample size making it necessary to be cautious while interpreting the results. Second, samples exceeding the internal control cut-offs were not excluded from the data since calculations were only performed on this training set and need further evaluation in an independent validation set. Third, since all women self-reported to have an HR-HPV infection in the previous six months, no documentation concerning their infection was provided to the study team and could therefore not be verified. We also did not receive information on whether the HPV infection was associated with a cervical intraepithelial lesion (CIN). Only 13 out of 25 women still had a detectable HR-HPV infection in at least one of the three collected samples. However, the objective of this study was not to compare urinary and cervical HR-HPV infections but to develop a reproducible optimal urine sampling and DNA extraction protocol. As this study is the first study to optimize the combination of FV urine collection volume, DNA extraction methods, and introduction of an internal control we are confident that these limitations will not have a major effect on the study outcomes.

## 4. Materials and Methods

### 4.1. Identification of the Internal Control Spiking Concentration

To investigate the accuracy of PhHV-1 DNA as internal control, four FV urine samples were used. All four healthy female volunteers collected ±25 mL of urine using a standard urine collection vial. These samples were divided into two falcon tubes; one tube containing 10 mL urine without UCM preservative, another one containing a total volume of 10 mL urine, and UCM in a ratio of 2:1 urine/UCM. DNA from PhHV-1 was extracted from 200 µL MEM culture medium of a PhHV-1 infected Crandell Rees Feline kidney cell line using the NucliSENS^®^ EasyMag^®^ (bioMérieux, off-board lysis protocol) and eluted in 100 µl. All eight samples were spiked with 25 µL of a 1:1000 dilution of this DNA eluate (stored at 4 °C). To investigate the quantity and stability of PhHV-1 DNA in the samples, 5 mL of each sample was used for DNA extractions (Figure A1). The remaining 5 mL of each sample was stored 7 days at 4 °C and an additional 5 days at room temperature after which DNA extractions using NucliSENS EasyMag on both FV urine concentrate after Amicon filtration of 4 mL (AM_EM) and 1 ml of neat FV urine (EM) were repeated for these samples. All samples were analyzed using semi-quantitative PCR (qPCR) for human DNA (GAPDH) and internal control DNA (PhHV-1) (see Section 4.5). Using this spiking volume, the initial Cq value for each UCM/FV urine combination would be 30–33, dependent on the DNA extraction method.

### 4.2. Study Population

In total, 25 women, age 19–62 years, were included between August and November 2019 (clinicaltrials.gov ID: NCT04480866) (Figure 1). Each woman self-reported to have an HR-HPV infection, which was identified during a gynecological visit in the previous six months. Informed consent was obtained from all women before sample collection.

### 4.3. Sample Collection and Storage

Women were notified about the study by email (University of Antwerp employees) or via social media. Women interested to participate in the trial first subscribed online via the Centre for the Evaluation of Vaccination (CEV) web page. Next, the study team contacted eligible women by phone and sent an additional e-mail to complete their registration if all inclusion criteria were met. Subsequently, a package with the information brochure, informed consent form, and collection devices with instructions for use was sent to the participants by postal mail. Each participant collected three consecutive samples at home, using prototype FV urine devices (Colli-Pee^®^, Novosanis) with collector tubes that differ in size, prefilled with 1/3 UCM (Novosanis) spiked with 25 µL of a 1:1000 dilution of PhHV-1 DNA extract as an internal control. This allowed us to collect an average of approximately 2.67, 6.67, and 13.33 mL urine in total volumes of 4, 10, and 20 mL respectively. Women were requested not to wash their genitals before collection, collect all samples on the same day, and not to urinate at least 2 h (h) before each collection. All samples were collected in random order, alternating the collection volumes to eliminate potential bias associated with sampling order. Earlier research already confirmed that morning FV urine does not contain higher (HPV) DNA concentrations than FV urine collected later during the day, eliminating this potential bias [32,39]. The same day of collection (or at the latest, one day after collection), the urine samples were sent back to the University of Antwerp via postal mail where they were aliquoted and stored at −80 °C (Biobank Antwerpen, Antwerp, Belgium; ID: BE 71030031000) prior to further analysis [40].

### 4.4. DNA Extraction from FV Urine Samples

Before every extraction, buffered aliquots of urine samples were thawed (after storage for 1–2 months). For this study, five different DNA extraction methods were used (overview in Figure 1). For four out of five methods (AM_EM, EM, CF_800__EM, and CF_3000__EM), the NucliSENS^®^ EasyMag^®^ (bioMérieux, off-board lysis generic protocol) was used and DNA was eluted in 65 µL for further analysis. The first DNA extraction method (AM_EM) used an in-house protocol [1]. Briefly, 4 mL of the FV urine aliquot was centrifuged at 3820 g for 20 min at 20 °C in an Amicon Ultra-4 50 K filter device (Merck Millipore, Belgium). Next, 2 mL NucliSENS Lysis Buffer (bioMérieux Benelux, Schaarbeek, Belgium) was added to the concentrate retained on the filter. After incubation of at least 10 min at room temperature, all material was transferred to the NucliSENS Lysis buffer vial and DNA extraction was performed on the buffered concentrate. For the second method (EM), direct DNA extraction was performed on 1 mL of the FV urine sample in 2 mL lysis buffer. The third extraction method (CF_800__EM) started with centrifuging 4 mL of the FV urine sample at 800 g for 10 min at room temperature. The pellet and 1 mL of supernatant were suspended in 2 mL lysis buffer and used for DNA extraction. The fourth extraction method (CF_3000__EM) used almost the same protocol as CF_800__EM, the only difference was centrifugation at 3000 g instead of 800 g. For the last extraction method (CF_800__QIA), 4 mL of the FV urine sample was centrifugated at 800 g for 10 min at room temperature. The pellet was resuspended in 200 µL of supernatant. DNA was extracted using the QIAamp DNA Blood Mini kit, according to the manufacturer’s protocol (Qiagen GmbH, Germany) and eluted in 65 µL H_2_O [1]. Different DNA extraction methods were compared for collection volumes; for 4 mL samples, extraction method EM was used; for 10 mL samples, extraction methods EM, CF_800__EM, and CF_3000__EM were used; for 20 mL samples, extraction methods AM_EM, EM, CF_800__EM, and CF_800__QIA were used (Figure 1).

### 4.5. Semi-Quantitative PCR for GAPDH and PhHV-1

All DNA extracts obtained from FV urine samples were analyzed using semi-quantitative PCR (qPCR) for human DNA (GAPDH) and internal control DNA (PhHV-1). This qPCR technique is based on TaqMan technology and performed with the LightCycler480 Real-Time PCR instrument (Roche Diagnostics, Machelen, Belgium) as described by Vorsters et al. (2014) [1]. Briefly, a 20-µL portion of the GAPDH PCR mixture containing 1× LightCycler^®^ 480 Probes Master (Roche Diagnostics, Machelen, Belgium), 0.5 μM concentrations of each primer, 0.1 μM concentrations of probe, and 5 μL of DNA solution was loaded into the LightCycler. Primers and probes for GAPDH are defined by Payan et al. (2007) [41]. For PhHV-1 qPCR was performed on 20 µL of the following mixture; 1 × LightCycler^®^ 480 Probes Master (Roche Applied Science, Belgium), 0.05 µM forward primer, 0.2 µM reverse primer, 0.1 μM probe, and 5 μL of DNA solution. PhHV-1 primers and probes are described by Van Doornum et al. (2002) [27]. The thermal cycles were as follows: an initial 10 min at 95 °C for FastStart Taq DNA Polymerase activation, followed by 45 cycles of 10 s of denaturing at 95 °C, 15 s of annealing at 60 °C. Both positive and negative controls were used on each run to confirm reproducibility.

### 4.6. HPV-Risk Assay

High-risk HPV testing was done using the HPV-Risk assay (Self-screen B.V., Amsterdam), which is a multiplex, real-time PCR assay targeting the E7 region of 15 (probably) HR-HPV types (HPV16, -18, -31, -33, -35, -39, -45, -51, -52, -56, -58, -59, -66, -67, and -68), and providing additional genotype information for HPV16 and HPV18 [42]. The assay includes amplification of the reference gene HBB to assess DNA quality and quantity. The assay was performed using 5 µL of the DNA extract according to the manufacturer’s instructions, on a Rotor-Gene Q MDx 5plex HRM instrument (Qiagen GMBH, Hilden, Germany). The Cq value cut-offs of the assay for calling a sample HPV-positive were not used. Samples were scored HPV positive when there was a Cq value for any of the HPV targets.

### 4.7. Semi-Quantitative PCR on Bisulfite Converted DNA

For testing the suitability of the DNA for quantitative Methylation-Specific PCR (qMSP), DNA extracted from FV urine was treated with bisulfite and amplified using primers and probes in the ACTB gene area containing at least three cytidines, as described previously [43,44,45,46]. Bisulfite-conversion was performed with the EZ DNA Methylation kit (Zymo Research, Orange, CA, USA) according to the manufacturer’s specifications. Standard DNA input for bisulfite-conversion was 250 ng. Elution was done with 12.5 µL M-elution buffer yielding 20 ng/µL bisulfite-converted DNA. For samples with insufficient DNA yield to accomplish an input of 250 ng (around 50% of the samples), a standard amount of 45 µL was used.

### 4.8. Statistical Analysis

For the statistical analysis, we used R statistical software version 3.6.0. The data were first checked for their normality using Shapiro–Wilk test. If the data were normally distributed, significant differences between different collection volumes and DNA extraction methods were examined using paired t-test, otherwise non-parametric Wilcoxon signed-rank testing was used. Statistical significance was defined as *p*-adjusted < 0.05 (using Holm–Bonferroni method for *p*-value adjustment). To analyze possible correlations between housekeeping genes (GAPDH, ACTB, HBB), Pearson correlation coefficients were calculated. To investigate HPV positivity agreement between different FV urine collection volumes and DNA extraction methods, Cohen’s Kappa (K) when maximum three conditions were compared and Fleiss Kappa values when more than three conditions were compared, were calculated. For the calculation of cut-off within the internal control data, the mean Cq plus two standard deviations were used [27]. To check whether there was an effect of time between collection and freezing on DNA yield for all endpoints, including internal control, we used a linear regression model on all EM data. 

## 5. Conclusions

In conclusion, we have evaluated a potential internal control of which the exact cut-off concentrations for sample validity will need to be determined in a validation study (NCT04530201). Good agreement between the three different FV urine collection volumes was found for all human endpoints. Because of the advantages of a lower collection volume in high-throughput testing and the highest percentage of HPV DNA-positive DNA extracts, the 10 mL collection volume is preferred. Overall, the preferred collection and processing combination within this study would be a 10 mL collection device, prefilled with UCM containing PhHV-1 as internal control, together with DNA extraction using 4 mL of the collected FV urine for centrifugation at 800 g whereof the pellet and 1 mL of supernatant will be used for NucliSENS EasyMag extraction (CF_800__EM).

## Figures and Tables

**Table 1:** HPV DNA Cq values (either HPV-31, -33, -35, -39, -45, -51, -52, -56, -58, -59, -66, -67, or -68) for all different first-void (FV) urine collection volumes and DNA extraction method combinations. Percentage of positive samples/extract is noted underneath. There were no significant differences between groups (*p* > 0.05). EM; EasyMag, CF; Centrifugation.

	HPV Cq Values							
	AM_EM	EM			CF_800__EM		CF_800__QIA	CF_3000__EM
	20 mL	4 mL	10 mL	20 mL	10 mL	20 mL	20 mL	10 mL
ID1	-	-	-	-	38.78	-	-	-
ID2	22.08	17.90	19.17	23.17	17.28	22.75	24.75	21.65
ID3	-	-	-	-	-	-	-	-
ID4	-	-	35.46	-	32.26	-	-	32.57
ID5	-	-	-	-	-	-	-	-
ID6	36.71	34.32	-	29.11	-	28.89	25.97	33.53
ID7	29.64	23.58	24.65	26.25	23.62	22.81	24.89	25.70
ID8	-	-	-	-	-	-	-	-
ID9	-	-	-	-	30.83	-	-	37.46
ID10	-	-	-	-	-	-	-	-
ID12	22.16	25.09	24.04	23.73	20.94	21.66	22.80	21.12
ID13	19.82	21.97	25.61	20.89	24.15	19.40	18.99	24.57
ID14	-	-	-	-	-	-	-	-
ID15	39.24	-	37.87	-	-	-	-	-
ID16	-	-	-	-	-	-	-	-
ID17	34.92	30.34	33.52	34.55	35.94	38.59	33.96	35.50
ID18	36.19	-	37.00	-	31.25	36.39	38.16	31.07
ID19	24.63	22.92	24.57	26.17	22.58	24.43	25.26	23.38
ID21	-	-	-	-	-	-	-	-
ID22	-	-	-	-	-	-	-	-
ID23	31.59	-	35.11	35.73	32.98	38.98	-	36.29
ID24	-	-	-	-	-	-	-	-
ID25	29.90	38.90	27.93	30.65	30.12	30.67	33.10	28.37
ID27	-	-	-	-	-	-	-	-
ID28	-	-	-	-	-	-	-	-
Percent positive (%)	44.00	32.00	44.00	36.00	52.00	40.00	36.00	48.00

## Data Availability

The data presented in this study are available from the corresponding author, L.T., upon reasonable request.

## Sample Availability

Not available.
